# Enhancement of the CAST Block Algorithm Based on Novel S-Box for Image Encryption

**DOI:** 10.3390/s22218527

**Published:** 2022-11-05

**Authors:** Rasha S. Ali, Omar Z. Akif, Sameeh A. Jassim, Alaa Kadhim Farhan, El-Sayed M. El-Kenawy, Abdelhameed Ibrahim, Mohamed E. Ghoneim, Abdelaziz A. Abdelhamid

**Affiliations:** 1 Electrical Engineering Department, College of Engineering, Al Iraqia University, Baghdad 10071, Iraq; 2 Department of Computer Science, College of Education for Pure Science (Ibn al-Haitham), University of Baghdad, Baghdad 10066, Iraq; 3 Department of Computer Sciences, University of Technology, Baghdad 10066, Iraq; 4 Department of Communications and Electronics, Delta Higher Institute of Engineering and Technology, Mansoura 35111, Egypt; 5 Computer Engineering and Control Systems Department, Faculty of Engineering, Mansoura University, Mansoura 35516, Egypt; 6 Department of Mathematical Sciences, Faculty of Applied Science, Umm Al-Qura University, Makkah 21955, Saudi Arabia; 7 Faculty of Computers and Artificial Intelligence, Damietta University, Damietta 34517, Egypt; 8 Department of Computer Science, Faculty of Computer and Information Sciences, Ain Shams University, Cairo 11566, Egypt

**Keywords:** CAST block, S-Boxes, 2D, 3D chaotic map

## Abstract

Background and Aim: due to the rapid growth of data communication and multimedia system applications, security becomes a critical issue in the communication and storage of images. This study aims to improve encryption and decryption for various types of images by decreasing time consumption and strengthening security. Methodology: An algorithm is proposed for encrypting images based on the Carlisle Adams and Stafford Tavares CAST block cipher algorithm with 3D and 2D logistic maps. A chaotic function that increases the randomness in the encrypted data and images, thereby breaking the relation sequence through the encryption procedure, is introduced. The time is decreased by using three secure and private S-Boxes rather than using six S-Boxes, as in the traditional method. Moreover, the CAST encryption algorithm was modified to be used on the private keys and substitution stage (S-Boxes), with the keys and S-Boxes of the encryption algorithm being generated according to the 2D and 3D chaotic map functions. The proposed system passed all evaluation criteria, including (MSE, PSNR, EQ, MD, SC, NC, AD, SNR, SIM, MAE, Time, CC, Entropy, and histograms). Results: Moreover, the results also illustrate that the created S-Boxes passed all evaluation criteria; compared with the results of the traditional method that was used in creating S-Box, the proposed method achieved better results than other methods used in the other works. The proposed solution improves the entropy which is between (7.991–7.999), reduces the processing time which is between (0.5–11 s/Images), and improves NCPR, which is between (0.991–1). Conclusions: The proposed solution focuses on reducing the total processing time for encryption and decryption and improving transmission security. Finally, this solution provides a fast security system for surgical telepresence with secure real-time communication. The complexity of this work needs to know the S-Box creation method used, the chaotic method, the values of the chaotic parameters, and which of these methods was used in the encryption process.

## 1. Introduction

Recently, both network communication and multimedia technologies have been developing rapidly [1]. Moreover, information across the world over the internet and other wireless networks has been experiencing exponential growth [2]. However, there have also been substantial data losses and damage because of increasingly sophisticated types of attacks and data breaches [3]. The main goal of cryptography is to keep and send the information to make only the authorized user be able to read and route it [4]. Cryptography is a science that applies complex mathematics to protect the privacy of information during communicating and storing [5,6]. It involves transforming information into apparently unintelligible garbage so that unwanted eyes are unable to comprehend it [7]. It is one of the most recent theories of physical physics—sometimes translated into chaotic theory—that deals with the subject of nonlinear (dynamic) moving sentences that exhibit a kind of random behavior known as schisms. This random behavior is either caused by an inability to determine initial conditions (butterfly effect) through the potential physical nature of quantum mechanics [8]. Moreover, the series invention of chaotic maps, such as tent, logistic, and Henon maps [9], has given researchers the ability to apply chaotic maps in a variety of fields [10,11]. Recently, chaotic with high sensitivity to initial conditions, non-periodicity, and strong periodicity have been commonly used in visual data security applications. There are several desirable features of chaotic-based cryptographic techniques, including sufficient protection and adequate processing time. These techniques involve two operations known as confusion and diffusion operations [12,13]. Therefore, chaos theory has been mixed up and embedded by research into other subjects [14,15].

**Figure 1:**
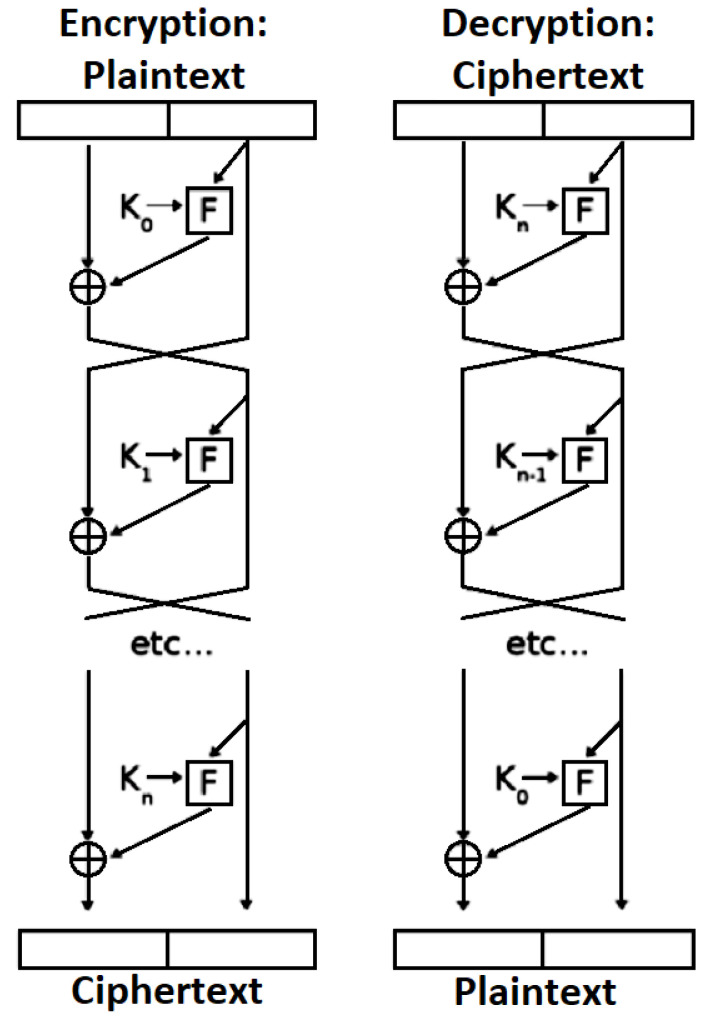
CAST-128 encryption and decryption.

Chaos models deal with unpredictable numbers, apparently random, but not necessarily so disorderly aura, tic, and irregular behavior [16]. The adopted use of identification procedure is based on nonlinear system synchronization theory [17]. Additionally, as a type of complex nonlinear system, chaotic systems have initial value sensitivity, pseudo randomness, and no periodicity, which thus provides a good match for the required characteristics of cryptography [18,19]. A chaotic sequence can be used as a random key such that the encryption impact achieved remains the same as the first time it was used. Hence, theoretically, encryption based on a chaotic sequence is difficult to break. Accordingly, chaotic encryption techniques are commonly used in the field of information security, especially for image encryption [20]. At present, most of the confusion and diffusion structure of image encryption algorithms is based on chaotic systems using chaotic sequences [21,22]. However, there are limitations regarding the computer word length, which can cause degradation in the chaotic dynamics, especially for a low-dimensional chaotic system [23,24]. Moreover, the security can be seriously affected by the limitations of chaotic encryption. Hence, with the aim of improving the security of chaotic algorithm, much research has been undertaken on hyper-chaos systems to ensure the complexity of the chaotic sequence. Nevertheless, nothing has been confirmed regarding to the encryption algorithm which is composed of a single chaotic map and cannot guarantee the security of an encrypted image [25,26]. In this research, a hybridization of a chaotic system based on a cast encryption algorithm is used to encrypt different types of digital images. CAST-128 is a procedure designed to be used as a symmetric encryption algorithm, which was developed by Carlisle Adams and Stafford Tavares [27,28,29]. CAST-128 is a classical feistily network, consisting of 16 rounds and operating based on 64-bit blocks of plaintext, with 64-bit blocks then being produced as a cipher text. It has been applied to deliver image encryption in an efficient manner, with the range of key size being from 40 bits to 128 bits in 8-bit increments. Figure 1 illustrates the procedure for both encryption and decryption in CAST-128 [30,31,32].

The main contribution of this work is to extend the area of research by enhancing the encryption algorithm of the CAST block cipher. To this end, 2D chaotic and 3D chaotic maps have been used to improve the CAST encryption algorithm. The 2D is used to generate dynamic keys and the 3D is used to generate dynamic S-Boxes. These are instead of using static keys and S-boxes of the traditional CAST algorithm. Since chaotic has features of generating random numbers without repetition and at a very high speed, it was used to solve one of the most important problems of traditional CAST. In addition, we are dealing with block by block in the encryption/decryption process instead of bit by bit as conducted in the traditional CAST encryption process.

This paper is divided into seven sections. In Section 2, one type of block encryption algorithm is introduced, while in Section 3, chaotic maps are discussed. Section 4 presents the methodology and, in Section 5, there is a discussion on Experimental Results relating to statistical tests, histogram analysis, information entropy, and encryption quality. The conclusion is provided in Section 6. This study aims to provide a secure algorithm for protecting all types of images transmitted over networks. The secure algorithm is applied based on chaotic systems and the CAST-128 block cipher algorithm. This algorithm is realistic according to three parts: first, the development of a 3D chaotic map for creating three S-Boxes, second, construction of 2D chaotic map functions for generating secure keys, and, third, modification of the CAST encryption algorithm for encrypting the images. A key aspect of chaos is changing in the initial values and parameters in such a way as to make keys and the S-Boxes difficult to guess. Research has consistently shown that the number of traditional S-Boxes is six; however, the time consumed during the installation stage is very high compared with the proposed method which involves just one S-Box.

## 2. CAST Block Cipher Encryption Algorithm

Carlisle Adams and Stafford Tavares (CAST): the name of a system designed by Carlisle Adams and Stafford Tavares, and this system has indicated their design system. The CAST system must be recalled using the images of randomness. A 64-bit block size and 64-bit key is a good example of the CAST algorithm, which provides the ability to use the input of combined S-boxes with 8-bit and 32-bit outcomes. The algorithm consists of eight rounds. The structure of these S-boxes is implementation-dependent and complicated. Because the CAST is a private method, it is used in a few areas, and the details of its S-Boxes are not announced. However, a simple brute force attack can break the CAST algorithm, so there is no other well-known method that has the ability to perform a successful attack. CAST is a standard encryption method certified by the Canadian government. The procedure of CAST encryption involves the following:

1. Read Plain text;
2. Divided Plain text into equal left and right blocks, thus providing 32-bits for each block;
3. Embedded Right block into the F function to generate a new right block, six S-Boxes, and XOR operations are involved in this function;
4. New left = old right;
5. The final left (L) and right (R) blocks will be exchanged and concatenated into the cipher form. Despite the decryption procedure going through the same steps that were mentioned above in the encryption procedure, the rounds (pairs of the subkey) have been used in reverse order to compute (L0, R0) from (R16, L16).

## 3. Chaos Theory

Chaos can be defined as an inherent random phenomenon, in numerous nonlinear dynamical systems for the both types, natural and non-natural. Nonlinear systems generate chaotic behaviors owing to several interior factors in their dynamics, which make them unique and hence of great interest [33]. Chaotic maps are classified into one-dimensional and multi-dimensional maps, according to their functionality, and one-dimensional chaotic maps are faster performing and less complicated for implementation than multidimensional ones overhead because of their high-dimensional structure. The main drawback with 1D shown, when short range of initial and input variables have been added, while multidimensional maps have been able to extend and have a wider range [34]. Recently, the sensitivity of chaotic and hyper-chaotic systems have been receiving increasing attention from researchers [35]. Notably, such systems can give more than one solution for a specific set of parameters and different initial values [36,37]. This nonlinear phenomenon has been referred to as coexisting attractors or multi-stability behavior. The sensors and devices needed to achieve security in transferring the sensing data and other security issues, such as communication (encryption, authentications and authorizations, secure protocols, secure routing, and other data and networking security) [38,39].

## 4. The Proposed Methodology

One of the most pressing concerns is data exposure to security attacks, even if they are encrypted. The present study is aimed at developing an encryption algorithm to increase security by raising the level of diffusion and confusion. That is, the objective is to contribute to this growing area of research by enhancing the encryption algorithm of the CAST block cipher. To this end, 2D chaotic and 3D chaotic maps have been used to improve the CAST encryption algorithm. Because the traditional CAST algorithm relies on a static method for generating keys and S-Boxes, so when we use Chaotic method, a variable dynamic generation of keys and boxes is provided, as well as Chaotic method known for its speed, which is one of the most important requirements currently for modern encryption algorithms. Since Chaotic has features of generating random numbers without repetition and at a very high speed, it was used to solve one of the most important problems of traditional CAST. Three S-Boxes have been created using a 3D chaotic map system, while a 2D chaos system has been utilized to generate the keys. Moreover, logical operation has been represented by applying the shift left and XOR operation. One of the most significant current discussions is that the x,y values result from the 2D chaotic map, while z comes from the 3D one. That is, these operations are dependent on using 3D to find the value of z. Hence, Z(i) is represented as the first digit number, and used for shifting the left of the original operations. The x-value in the 2D chaotic map is used to obtain the integer number from the right float number, whilst the value of y is used with the left integer digit and the values of the 2D chaotic map are used in the XOR operation. For example, R = R(i − 1) XOR x and L = L(i − 1) XOR y.

**Figure 2:**
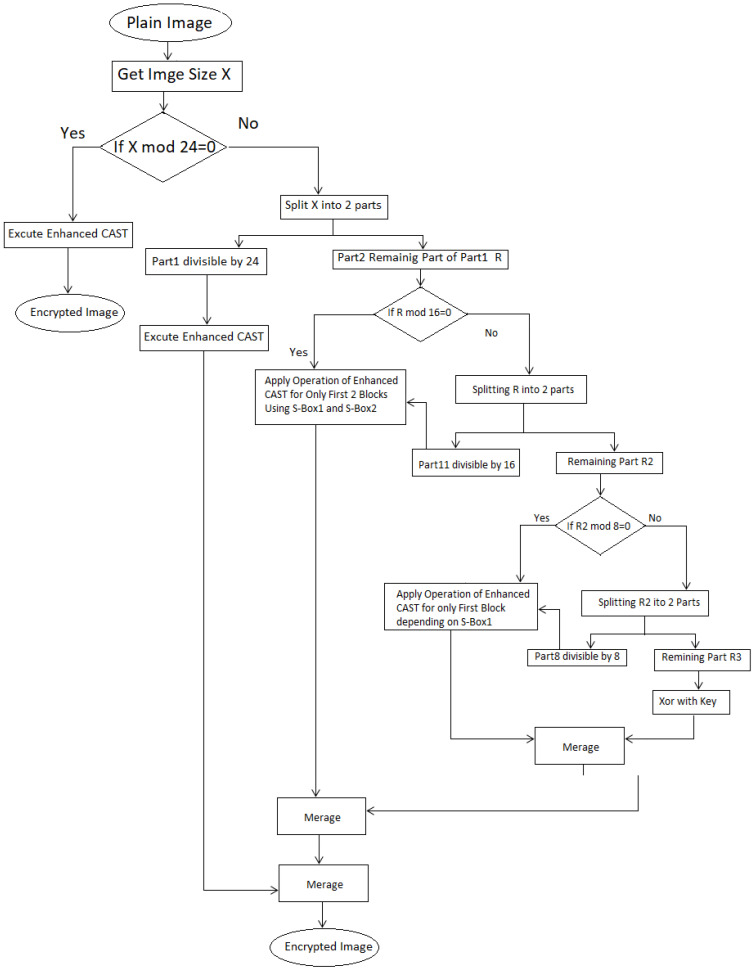
Flowchart of the proposed encryption method.

The encryption algorithm involves taking 768 bits and dividing them into three blocks, each containing 256 bits. Following this, these blocks represent the colors of 32 pixels, with each pixel including three colors of 8 bits per color. The 768 bits is calculated by multiples [(32 pixels) × (3 color) × (8 bit)], hence the block size is represented in 32 pixels. The traditional CAST is then applied to each block, with one significant difference, which is using one S-Box for each block with private keys. Moreover, the S-Boxes are generated based on 3D chaos (the first cell in an S-Box is based on the first left digit number from x,y,z). For example, let i = 0, x = 21,435, y = 66,754, and z = 8742, then s-box(0) = 260 mod 255, which is equal to 05. The same procedure is used to generate the remaining 255 cells in the S-Box. Moreover, x and y, the values of 2D chaos, are used for XOR operations, thus improving the CAST algorithm. The second S-Box is created by going in the same way as the first, except that the initial values are different and the same procedure is applied to the third S-Box (Section explains the generation of S-Boxes in further detail). Figure 2 illustrates the flowchart of the proposed encryption algorithm.

### 4.1. Key Generation

The keys are generated by applying the 2D logistic map approach, and their different values depend on the image size (number of pixels). The key generation algorithm is presented in Algorithm 1, and the following equations are applied throughout the stage as key generators in the proposed algorithm [40]. Equations of the 2D logistic map are presented in the following:

(1) $$x_{i+1}=r\left(3y_{i}+1\right)*x_{i}*\left(1-x_{i}\right)$$

(2) $$Y_{i+1}=r\left(3x_{i}+1\right)*y_{i}*\left(1-y_{i}\right)$$

**Algorithm 1** Key generation algorithm1:**Input**: r,x(0),y(0)2:**Output**: *N* different values as user request3:**Begin**4:**Step 1**: Calculate x(i+1) by applying Equation (2)5:**Step 2**: Calculate y(i+1) by applying Equation (3)6:**Step 3**: Convert x,y values to integers by removing the numbers that exist before comma (integer part number), such that let x(1)=0.3432, and y(1)=0.6547 the integer values, x(1)=3432 and y(1)=6547 // only real values are used in the generated keys. The real values are extracted by using the fractional part number (after the comma)7:**Step 4**: Repeat steps 1, 2, 3 and 4 n times and to obtain the generated keys8:**Step 5**: Get the distinct values of Step 49:**Step 6**: Fill the key array with the values generated in Step 5

### 4.2. S-Box Creation

In this proposed method, the S-Box creation process involves a 3D logistic map technique to improve the CAST encryption algorithm. The steps of the S-Box creation process are listed in Algorithm 2. The main parameters of these S-Boxes depend on using x, y, and z, which are the values of the 3D chaotic function. In addition, the S-Box inverse is configured according to the parameters and processes installed in the S-Box. There are two goals behind using the chaos theory; firstly, it is to generate random numbers without redundancy and, secondly, to increase the number size. Therefore, this is a significant requirement in terms of data security (raise the randomness at a high speed). The following equations illustrate the S-Box creation process used in the proposed approach [40]. Equations of the 3D logistic map are presented in the following:

(3) $$x_{i+1}=r*x_{i}\left(1-x_{i}\right)+\beta *y_{i}^{2}*x_{i}+\alpha *z_{i}^{2}$$

(4) $$Y_{i+1}=r*y_{i}\left(1-y_{i}\right)+\beta *z_{i}^{2}*y_{i}+\alpha *x_{i}^{2}$$

(5) $$z_{i+1}=r*z_{i}\left(1-z_{i}\right)+\beta *x_{i}^{2}*z_{i}+\alpha *y_{i}^{2}$$

**Algorithm 2** S-Box generation algorithm1:**Input**: β=1.1,α=0.8,r=4,x(0)=0.9,y(0)=3.9,z(0)=2.92:**Output**: 256 different values (to create a new A-Box)3:**Begin**4:**Step 1**: Calculate x(i+1) by applying Equation (3)5:**Step 2**: Calculate y(i+1) by applying Equation (4)6:**Step 3**: Calculate z(i+1) by applying Equation (5)7:**Step 4**: Convert x,y,z values to integer by removing the numbers that are exists before comma (the integer part number), such that let x=0.12345, y=0.876 and z=0.6542 the integer values for x,y and *z* equal to (12,345,876 and 6542), respectively. // only real value was used in the generated keys. The real values extracted by cutting the fractional part numbers (after comma)8:**Step 5**: Generate the first cell (position (0,0)) in S-Box values by taking the first digit number from each of x,y and *z* mod 255. Like S-Box(i) = 186 mod 255, which is equal 186. // and the 186 was taken from the above example by taking the first digit value from each x,y and *z*, respectively9:**Step 6**: Repeat steps 1,2,3 and 4 n times and then obtain the distinct 256 numbers from the generated values10:**Step 7**: Convert all S-Box values obtained from Step 6 to a hexadecimal number11:**Step 8**: Fill the S-Box table with the values generated by Step 7.

### 4.3. Enhanced CAST Encryption Algorithm

Recall, in this proposed technique, that one of the main objectives is to encrypt the digital image in an effective way based on the CAST encryption algorithm. Moreover, two types of chaotic maps have been used, 2D and 3D chaotic. The main purpose of the 2D chaotic map function is to generate the keys, while a 3D chaotic map function is to generate the S-Boxes. To start with, input the plain image is inputted and then split into blocks of size 768 bits (explained before in Section 4), which are divided into three smaller blocks, each constituting one S-Box. Each block is then split into two parts: left (Li) and right (Ri). Hence, three operations are carried out on the right part: S-Box, XOR with the X(i), and XOR with the left part (Li). The value of d comprises the outcomes of the previous step (the three operations). The result produces a new right (Ri + 1) part, while the new left is calculated by [left (Li + 1) = R(i) XOR Y(i)]. One of the most significant points regarding this approach is that, when the remaining number of pixels is less than 8, then these remaining pixels are encrypted using the XOR operation with the keys that have been generated before. If the number of pixels is more than or equal to 8 and less than 24, then cut the part which is divided by 8 or 16, after applying the enhanced CAST on this part by using only the operations in the first and second part. In contrast, XOR operation with the key is applied on the remaining part as aforementioned. The main modification in the proposed method pertains to creating left and right parts, so, instead of using one key for encryption, two keys are used, which increases the complexity of the encryption algorithm. Another contribution of this approach is using a new S-Box generation method using the 3D chaotic function built into the S-Box generation process, instead of using the traditional CAST S-Box. Hence, both 2D and 3D chaos theory have been used in the modified procedure to encrypt the image. The decryption procedure goes through the same steps of the encryption procedure in reverse. Figure 2 illustrates the flowchart of the proposed encryption method and the steps of the proposed method are listed in Algorithm 3.
**Algorithm 3** Enhanced CAST encryption algorithm1:**Input**: Plain Image, X,Y,Z,α,β2:**Output**: Encrypted Image3:**Begin**:4:**Step 1**: Find the key values by applying Algorithm 15:**Step 2**: Calculate S-Boxes values by applying Algorithm 26:**Step 3**: Select the 32 pixels called it S (768 bits)7:**Step 4**: Dividing S into three blocks as B1, B2 and B3 // each block 256 bits8:**Step 5**: For *J*, steps from 6 to 9 are applied to each block (B1, B2 and B3)9:**Step 6**: Converting the block to binary10:**Step 7**: Divide the block into left (Li) and right (Ri) halves, with each block including 128 bits11:**Step 8**: For I from 1 to 16, compute Li and Ri as follows:$L_{i}=R_{i-1}$ XOR $Y_{i-1}$$R_{i}=S-Box_{j}\left(R_{i}-1\right)$ //where j=1 to 3, $SBox_{j}=SBox_{1},SBox_{2},$ and $SBox_{3}$ based on block number$R_{i-1}=R_{i}$$R_{i}=R_{i-1}$ XOR $L_{i-1}$ XOR $X_{i-1}$**Next I****Step 9**: Replace the final and concatenate to form the cipher image12:**Next J**

## 5. Experimental Results

**Figure 3:**
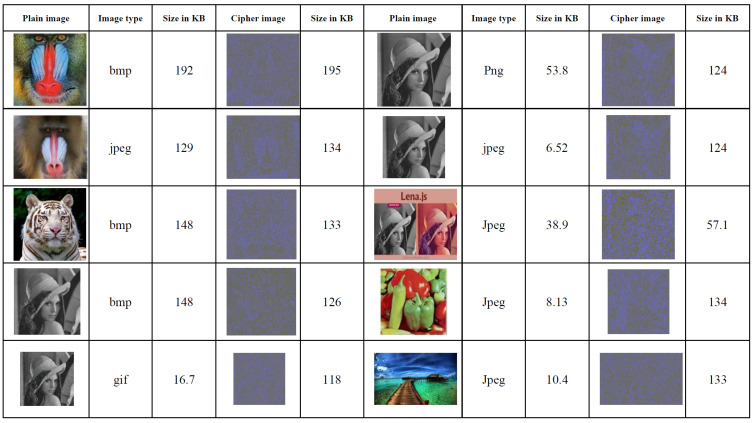
The original images in columns (1st, 6th) and the ciphered images in columns (4th, 9th).

An experimental investigation was conducted to explore the robustness of the proposed approach, the results of which are presented in this section. Vb.net 2013 has been used to implement and simulate the proposed method. Furthermore, the quality of the system has been tested by applying it to different sizes and types of images, such as JPEG, Ping, Bmp, etc.; thereby, the variety of images makes a proposed system offer users more flexibility. For example, suppose user A has several types of images that must be encrypted; if the proposed system works with just one type image, then the user is unable to encrypt all the images. Figure 3 illustrates the images before and after the encryption process (the original and ciphered images). More images included in the conducted experiments are shown in Appendix A. We performed a test on 18 different images types and some of them includes surgery images. Because of the proposed method having strong security features such as high speed and strong security, it is more suitable to be applied for securing medical images. The properties of the proposed encryption, security analysis, and statistical tests are explained and results provided in Section 5.1 and Section 5.2.

The research results were for applying proposed work on slim mini-computer with properties of Intel(R) Core(TM) m7-6Y75 CPU, 1.20 GHz, 1.51 GHz, 8 GB RAM, and a 64-bit operating system, and when applying it on another laptop with properties: Corei7, 4th generation, 8 GB RAM, and 64-bit operating system, the processing is three times faster. The vb.net 2015 was used in programming the proposed methods.

### 5.1. Security Scheme Discussion

1. Security Analysis: Strong evidence was found regarding how the proposed keys generation based on a 2D chaotic map is robust for encryption and decryption due to their being very difficult to guess. A further powerful point pertaining to the keys is that, if an adversary obtains part of them by using a brute force attack, then he or she does not have the ability to generate or obtain the other keys because the key generation algorithm is very sensitive to the values that have been entered as initial values and hence he or she cannot recognize the process involved and the initial values needed to be known, and, consequently, the proposed method is resistant to brute force attacks.
2. Chosen cipher text attack: In addition to a block cipher method having been used in the proposed method, the stream cipher technique has been included by applying XOR operation. A cipher-text attack has difficulty succeeding as, in each round of the stream cipher, the keys are changing and there is a different key for each block. Furthermore, the attacker needs to have prior knowledge of the original and encrypted text, without which it is impossible to obtain the plaintext.
3. Key space analysis: The key space is the set of all possible security keys that can be generated by using the proposed method (2D chaotic function). The proposed key generation method means that the number of keys is equal to the number of image pixels. The 2D chaotic function means that, if the height of the image = 255 and width = 255, then the proposed method creates 255 × 255 keys for x,y, respectively, thus providing very powerful protection against brute force attacks. Additionally, the proposed method is generating distinct keys without redundancy in the values of keys, which increases the security of the proposed method.

### 5.2. Statistical Tests

#### 5.2.1. Information Entropy Analysis

Information entropy has been used to measure the uncertainty of the information source [41]. Clearly, the value of ideal information entropy for the eight bits gives eight randomly messages. The entropy of information source is represented by H(X) in Equation (6) and hence $X=(x_{0},x_{1},\ldots ,x_{L-1})$ [42]:

(6) $$H\left(X\right)=-\sum_{i}p_{i}log\left(p_{i}\right)$$

The results of this measurement are shown in Table 1, where the proposed system can be seen as nearly achieving the theoretical value of 8.

#### 5.2.2. Histogram Analysis

**Figure 4:**
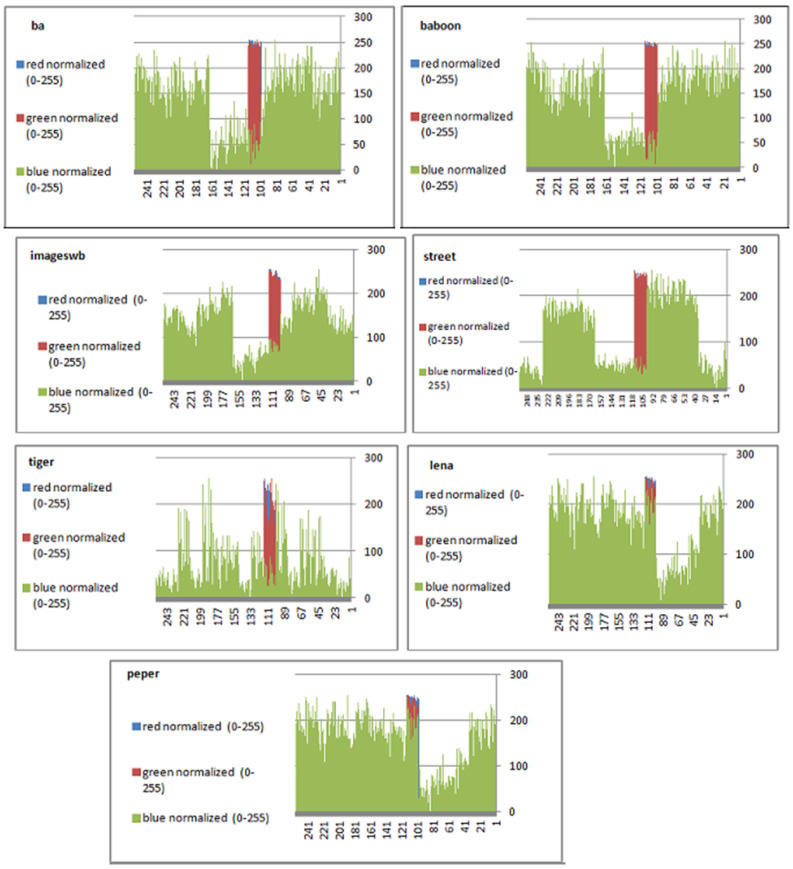
Histogram of the plain image.

**Figure 5:**
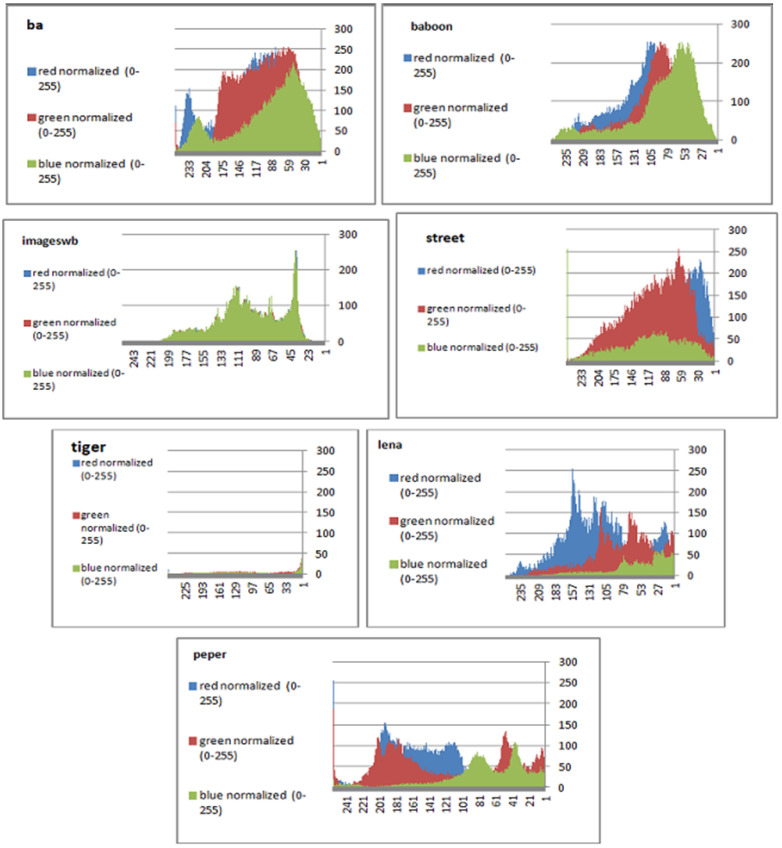
Histogram of the cipher image.

**Figure 6:**
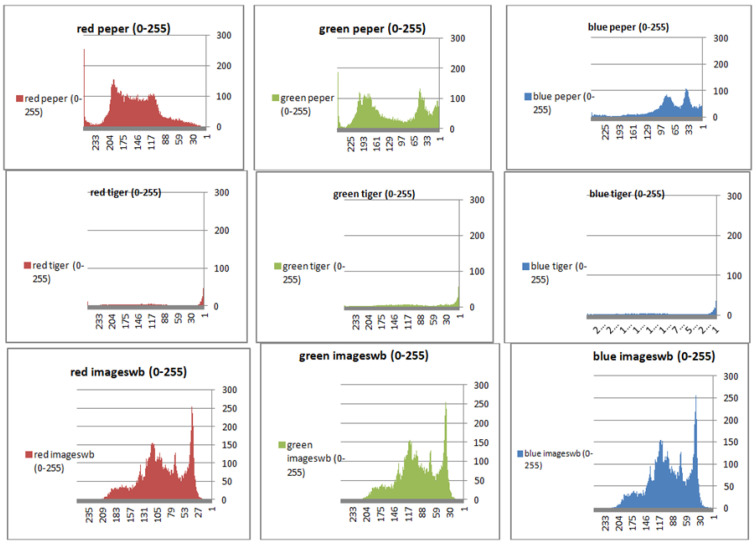
Histograms (red, green, and blue) for the original images.

**Figure 7:**
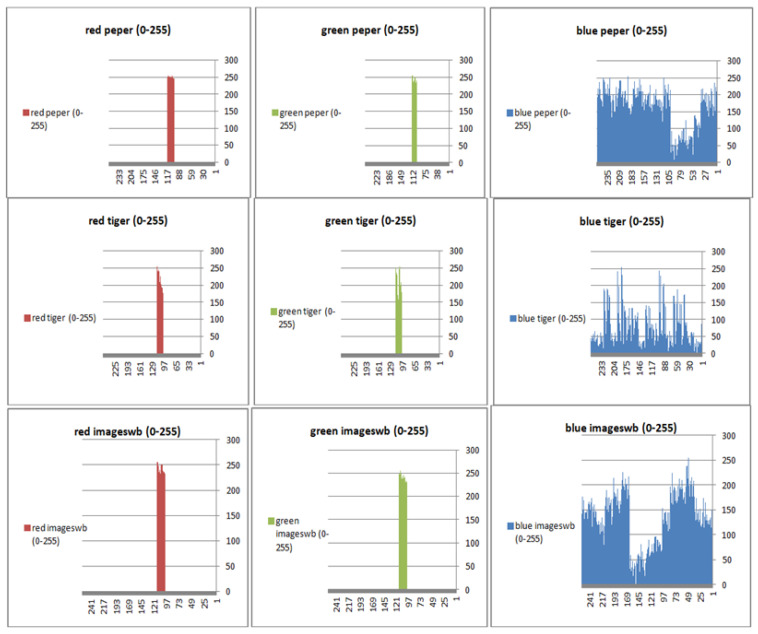
Histogram (red, green, and blue) for encrypted pepper images.

To prevent the information from leaking and aggressive attacks, both the encrypted and original images must not have any statistical similarity. To prove these objectives, histograms have been used to illustrate the distribution of pixels for each image in terms of the number of brightness pixels. A significant difference between the original and encrypted images can clearly be seen in the histograms, thus indicating that there is no statistical similarity. If an adversary tries to use histogram analysis of the encrypted image, then he/she is not able to obtain any information from the original image. Furthermore, a good indicator regarding of the success of the cryptosystem is that the appearance of a histogram for the cipher image must be evenly distributed, and this means that an attacker cannot extract the statistical features of the original image from a cipher image. Figure 4 shows the histograms of the plain images, while the cipher images are illustrated in Figure 5. It is evident that the proposed encryption method makes the information of the histograms safely obscured. Figure 6 represents the red, green, and blue of original images, while Figure 7 shows the cipher image in the channel.

In addition, we also conducted the chi-square test to additionally evaluate the uniformity of the histogram as shown in Equation (7):

(7) $$\chi^{2}=\sum_{L=0}^{255}\frac{{\left(o_{L}-e_{L}\right)}^{2}}{e_{L}}$$

where *L* is the intensity level, and $o_{L}$ and $e_{L}$ are the observed reference and the expected reference of the gray level in the encrypted image, respectively. Table 2 shows the chi-square test results of the plain encrypted images. The smaller the chi-square value, the more uniform the pixel distribution and thus the higher the security.

**Chi-square measurement:** Uniformity caused by the encryption algorithm is justified by the chi-square test. Relatively uniform distribution in cipher-image histogram points out the good quality of the method. To prevent the leakage of information to attackers, it is important to ensure that encrypted and original images do not have any statistical similarities. The histogram analysis clarifies how the pixel values of the image are distributed.

#### 5.2.3. Encryption Quality

**Figure 8:**
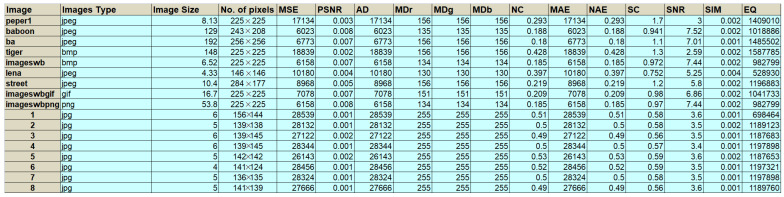
Results of statistical tests for the proposed method.

Encrypted images affect the image by creating a huge changing in the pixels. Thus, the result of image encryption makes the pixels completely differ from an original image, and these changes could be random (irregular). Additionally, when the changing of values is increasing, then the encryption algorithm is more effective. The results, as shown in Figure 8, indicate that the proposed algorithm delivers good encryption quality ranging between 528,930 and 1,587,785.

#### 5.2.4. Peak Signal-to-Noise Ratio (PSNR) and Mean Square Error (MSE)

The aim is to decrease the PSNR to the cipher image, for there are significant positive results when the PSNR values are low. However, the PSNR for steganography should be increased. Furthermore, another measurement has been taken is the MSE, which for steganography should be decreased, but, for a ciphered image, it should be increased. The results, as shown in Figure 8, indicate that these two requirements are achieved:

(8) $$PSNR=10log_{10}\left(255^{2}/MSE\left(f,g\right)\right)$$

#### 5.2.5. Similarity Measurement (SIM)

This measurement is used to ascertain the similarity between the original and ciphered image. For the encryption process, this measure should be decreased, The results emerging from the data for this measurement are shown in Figure 8. That is, Equation (3) has been used to calculate the similarity between the original and cipher image, with good results having been achieved in terms of increasing the ratio. The time for both encryption and decryption is enhanced through application of the proposed system, and Table 3 illustrates the time required for the encryption and decryption operations for different sizes of images:

(9) $$SIM=\frac{\sum \sum f_{i,j}*f^{\prime }\left(i,j^{\prime }\right)}{\sum \sum f_{i,j}*f^{\prime }\left(i,j\right)}$$

#### 5.2.6. Correlation Analysis

The correlation between the original and encrypted images was tested. When the values of the correlation are low, this is a good indicator that there is no relation between the two images. Correlation values have been calculated and measured for the proposed method based on Equation (9), with results showing that values are good in that they are less than zero, as can be seen in Table 3. Then, the correlation coefficient calculates $r_{x,y}$ by using Equation (9). These results mean that there are no correlations between the original and encrypted images:

(10) $$r_{x,y}=\frac{n(\sum xy)-(\sum x)(\sum y)}{\sqrt{\left[n\sum x^{2}-{\left(\sum x\right)}^{2}\right]\left[n\sum y^{2}-{\left(\sum y\right)}^{2}\right]}}$$

The correlation coefficient is the specific measure that quantifies the strength of the linear relationship between two variables in a correlation analysis. One significant criterion is the correlation coefficient that is used for the statistical analysis of encrypted images. The correlation coefficient assesses the correlation between two adjoining pixels in an image. Generally, correlation measures the degree of similarity between two pixels. In general, a high correlation exists between the adjacent pixels of an image, whereas a poor correlation should be there between the neighboring pixels of the corresponding cipher image.

#### 5.2.7. Number of Pixels Change Rate (NPCR)

The NPCR, as shown in Table 3, is used to evaluate the sensitivity of the key and the plain-image. It is calculated by the following:

(11) $$NPCR=\sum_{i=0}^{H}\sum_{j=0}^{W}\frac{D(I,j)}{T}*100\%$$

where

$$\begin{array}{c} D\left(i,j\right)=\left\{\begin{array}{cc} 0 & c_{1}\left(i,j\right)\neq c_{2}\left(i,j\right)\\ 1 & c_{1}\left(i,j\right)=c_{2}\left(i,j\right) \end{array}\right., \end{array}$$

*H* is the weight of image, $c_{1}$ and $c_{2}$ are the encrypted images, and symbol *T* denotes the total number pixels in the cipher image. NPCR is widely used in security analyses in terms of differential attacks and applied on the image encryption. It focuses on the absolute number of pixels which have changed the values in differential attacks [43]. Table 3 shows evidence of how the proposed method being strong was found when the values of NPCR were >99% for each color component image, and these values were obtained from ten different images. Experimental results illustrate that the estimated expectations and variance of NPCR are very close to the theoretical values. Hence, the proposed encryption scheme is resistant against differential attacks.

### 5.3. Generated S-Box Performance Analysis

**Figure 9:**
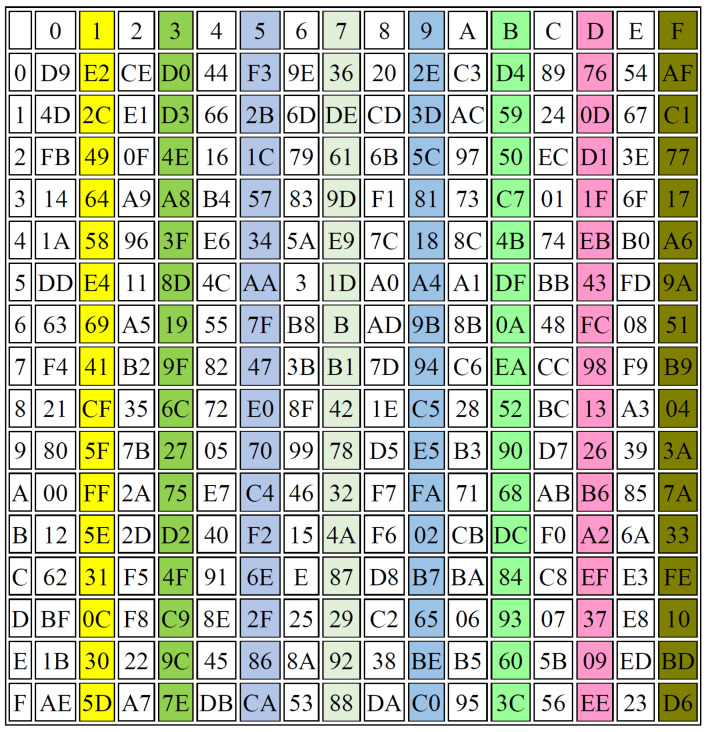
S-BOX1, r = 4.

**Figure 10:**
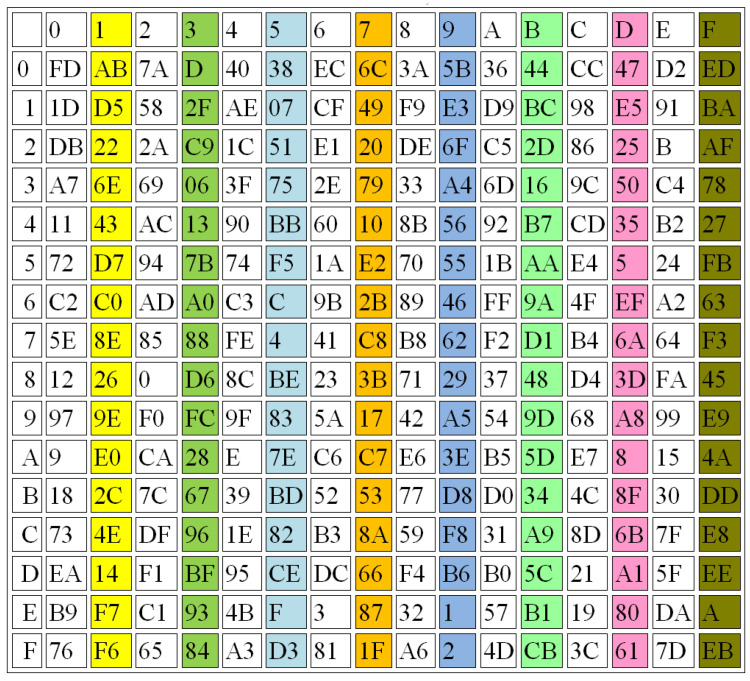
S-BOX2, r = 4.001.

**Figure 11:**
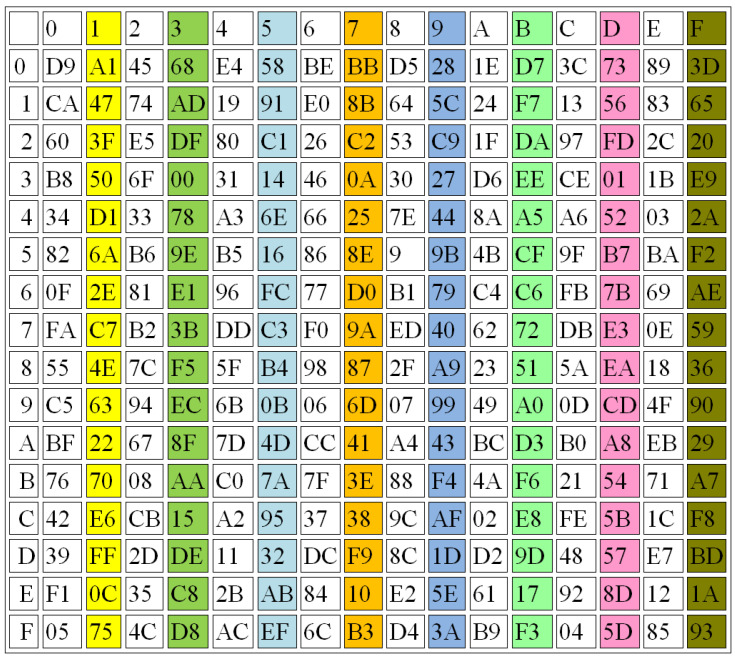
S-BOX3, r = 4.0000001.

In this section, several performance evaluations are applied to prove the effectiveness of the proposed S-box design, including balance, strict avalanche criterion (SAC), and the output bit independence criterion (BIC). Figure 9, Figure 10 and Figure 11 illustrate some examples in generating the 3 S-Boxes using different r values.

Figure 9, Figure 10 and Figure 11 show that the proposed method achieves good results when the three S-Boxes are compared together, while Table 4 shows the average differences between them.

Balanced: The three S-Boxes are balanced, if they have the same number of ones and zeroes, which is one of the important features relating to them. The comparison results for the proposed S-box design and the other existing methods are illustrated in Table 5 and Table 6 regarding the balance criteria.

There is a significant difference between the traditional methods and the proposed one. When the results of this study are compared with the findings of previous work, it emerges that the proposed method gives a better balance than the traditional methods used for generating the S-Boxes, as shown in Table 7. The proposed method includes the numbers of 1s that are equal to the numbers of 0s in a range of 93.7% to 100%, while the other methods recorded ranges of 1s and 0s between 87.5 and 98.4 [35] and from 92.2 to 98.4 [36].

- Completeness: The S-Boxes are complete, if every output bit is dependent on all the input bits. The function Y is considered complete, if there is at least one pair of plain text vectors (*z* and $z_{i}$), such that: (*z* and $z_{i}$) are n bit vectors that are variant in just one bit *i* and y(z) and $y(z_{i})$ vary at least in bit *h*, for all *i*. For example, one S-Box is generated from parameters β=1.1,α=0.8,r=4,x(0)=0.9,y(0)=3.9,z(0)=2.9 and another one is constructed using parameters β=1.1,α=0.8,r=4.001,x(0)=0.9,y(0)=3.9,z(0)=2.9. The results of these examples are shown in Figure 10 and Figure 11, respectively.
- Avalanche Criterion (AC): A block cipher is considered to detect the effect of an avalanche when a single bit of the input has been changed; then, a huge difference occurs in the output. The range of AC should be between 0 and 1, with the best value being 0.5, which results in an S-Box satisfying the avalanche criterion. The avalanche of the transformation function for the S-Box can be obtained using the following equation [44,45]:
  (12) $$Avalanche-Effect=\frac{Number-of-Fliped-Bits-in-(output-Cipher)}{Number-of-all-Bits-in-(output-cipher)}$$

For example, let K be the input used for S-Box1, while K1 is another input also used for S-Box1, but with a small change (only one bit will be changed). For example, let K = “B” and K1 = “C”

Binary (K) = “01000010” and Binary (K1) = “01000011”. The output from the S-Box (Table 7) will be in Hex:

Output (K) = “96” in Binary = “10010110”

Output (K1) = “3F” in Binary = “00111111”

The difference between output (K) and output (K1) is cleared in 5 bits; then, the AC, according to Equation (3), is: AC = 5/8 = 0.625

Let K = “E” and K1 = “F”, Binary (K) = “01000101” and Binary (K1) = “01000110”, output from S-Box:

Output (K) = “34”in Binary = “00110100”

Output (K1) = “5A” in Binary= “01011010”

The difference between output (K) and output (K1) is cleared in five bits; then, the AC, according to Equation (3), is: AC = 5/8 = 0.625.

The results from the above examples show that the proposed method achieves a near-to-ideal AC value; then, the avalanche criteria are achieved. Table 6 represents the count of variable bits when changing one bit per letter (the example shown is for English letters). The following example is a sample of two English letters to show how the calculation above is executed.

For example, B and Z are characters in the AC testing process: as presented in Table 8.

- **The study presented in [35]**:
  ASCII (B) = 66 Hex (65) = 42 Binary (66) = 01000010 output of S-Box (42) = 83 binary (output) = 10000011 changing one bit of actual data= 01000011this string in hex = 43 output of S-Box (43) = 56 in binary= 01010110 = 5/8 = 0.625.
  ASCII(Z) = 90 Hex(90) = 5A Binary(90) = 01011010 output of S-Box (5A) = AC binary (output) = 10101100 changing one bit of actual data = 01011011 this string in hex = 5B output of S-Box(5B) = 64 in binary = 01100100 AC = 3/8 = 0.375.
- **The study presented [36]**:
  ASCII (B) = 66 Hex (66) = 42 Binary (66) = 01000010 output of S-Box (42) = BD binary (output) = 10111101 changing one bit of actual data = 01000011 this string in hex = 43 output of S-Box (43) = 8D in binary = 10001101 AC = 2/8 = 0.25.
  ASCII (Z) =90 Hex (90) = 5A Binary (90) = 01011010 output of S-Box (5A) = 97 binary (output) = 10010111 changing one bit of actual data= 01011011 this string in hex = 5B output of S-Box (5B) = 8c in binary= 10001100 AC=5/8=0.5.
- **The proposed methodology**:
  ASCII (B) = 66 Hex (66) = 42 Binary (66) = 01000010 output of S-Box (42) = 96 binary (output) = 10010110 changing one bit of actual data = 01000011 this string in hex = 43 output of S-Box (43) = 58 in binary = 01011000 AC = 5/8 = 0.625
  ASCII = 90 Hex(90) = 5A Binary (90) = 01011010 output of S-Box (5A) = A1 binary (output) = 10100001 changing one bit of actual data = 01011011 this string in hex = 5B output of S-Box(5B) = DF in binary = 11011111 AC = 6/8 = 0.75.

**Bit independence criterion**: The bit independence criterion (BIC) was established by Webster and Tavares [44] and is another method used to evaluate the independent change of the output bits for S-boxes when a single input bit has been changed. In other words, the BIC checks whether the set of vectors generated with the reverse bit of a plaintext is independent from all avalanche variable sets.

**Nonlinearity**: It is one of the most important features in the S-box value evaluation criteria [28]. Mathematically, it can be defined by:

(13) $$S_{\left(f\right)}\left(w\right)=\sum_{w\in GF(2^{n})}{\left(-1\right)}^{f(x)⨁x.w}$$

where the dot product between *x* and *w* is defined by:

(14) $$x.w=x_{1}.w_{1}⨁w_{2}.w_{2}⨁\ldots x_{n}.w_{n}$$

Thus, the nonlinearity is calculated by:

(15) $$N_{f}=2^{n-1}\left(1-2^{n}\max_{w\in GF(2^{n})}\left|S_{\left(f)(w\right)}\right|\right)$$

Nonlinearity values of the generated S-Box by the proposed algorithm are 108, 110, 106, 112, 108, 106, 112, and 106. However, Table 8 compares the nonlinearity of the proposed S-box design with other existing S-box methods. It can be seen from Table 9 that the average value of the proposed S-box is greater than other existing methods. Based on this evaluation, it can be concluded that the proposed S-box design has good nonlinearity performance.

By comparing the results of this research with those achieved by authors in [16], we notice that the results for the proposed work were better because the correlation of the reference [16] ranged between 0.0082 and 0.0032, while the results of the proposed work ranged between –0.0001 and –0.0004, and this means that there is no correlation between the original images and the encrypted images. On the other hand, the entropy is that the results of the reference [16] of the image (lena) were (7.990), and the results of the proposed work were higher (7.991), and this also means that we reached better results with the encryption process as well. In addition, when comparing the results of [44], we can note that the proposed method achieved better results because of the rate for correlation coefficient of reference [44] being about 0.00123 for the Lena image and 0.0045 for the Baboon image, while the proposed method is –0.0001 for the Lena image and –0.0002 for the Baboon image. There is another comparison for the reference and the proposed work the entropy for (Lena and Baboon) image in reference [44] is (7.903 and 7.902) and for the proposed work is (7.991 and 7.998) and the appropriate value must be near 8. There are another comparisons between reference [45] and the proposed work. These are according to PSNR, MSE, and EQ evaluation measures: (1) In the reference [45], these are (0.0047, 10150.39, and 133266) for the Peper image, and (0.0053, 9016.81, and 12609) for the Lena image. (2) However, the PSNR, MSE, and EQ evaluation measures for the proposed results are (0.003, 17134, and 1409010) for the Peper image, and (0.004, 10180, and 528930) for the Lena image. For the encryption process, the PSNR must be decreased, while the MSE and EQ must be increased and EQ must range between 528930 and 1587785. From this point, we note that the proposed method achieved better results and the EQ of [45] fewer than the appropriate value. Since the results of the proposed S-Box were better when compared with the other previous works, the encryption results will also be better when compared to the earlier works.

In addition to the previous comparisons, there are other comparisons between references [50,51]. For the proposed work for the time correlation coefficient measures in [50], the time range is 8–78 s for images of size 128 × 128 pixels, while, for the proposed work, the time range is 0.5–11 s for images of different sizes bigger than 128 × 128). This means that the proposed work improved the previous works by decreasing the consumed time, and this is one of the most important issues in the encryption process. On the other hand, when comparing the proposed work with reference [50] for the correlation coefficient measurement, it is shown that the correlation coefficient [50] ranged between 0.0167 and 0.0457 for original CAST, and, for the Modified CAST of reference [50], the correlation coefficient ranged between 0.0046 and 0.0313. However, for the proposed work, the correlation coefficient ranged from –0.0004 to –0.0001. This result means that the proposed work achieves better results by breaking all the relations between the original and ciphered images.

### 5.4. Randomness Test for NIST Statistical Test

As illustrated in Table 10, the sixteen samples of the statistical NIST test are applied to the output results of the proposed approach. These measurements look for different forms of non-randomness, entropy, frequency, and runs tests. This may be found in a sequence. All results are passed successfully to the NIST test. Table 9 shows a comparison of the NIST test among the ciphertext output results of the proposed method and the ciphertext output results of the ref. [52] algorithm. In addition, the proposed procedure divides the images into blocks by converting it to binary numbers.

NIST Test Criteria is another evaluation criterion applied in addition to the previously discussed evaluation measurements. The NIST test is used for measuring the security of the proposed work, and the results show that the proposed method has good security performance.

## 6. Conclusions

In this study, a CAST block cipher algorithm with chaos theory has been proposed for use in encrypting any type of image. Furthermore, various types of statistical tests have been performed to evaluate and thus prove the effectiveness of the proposed algorithm. Therefore, according to the evaluation tests, findings of the proposed system have passed all the evaluation measurements. Two types of chaotic maps have been used in the proposed system: 2D and 3D. The 3D chaos map was used to generate the S-Box, while the 2D chaos map was used for the key generation process. The proposed image cipher has been rated with a high encryption level, as requiring less computations, and it was indicated with a high sensitivity using a secret key; hence, each user has their own secret key which was represented in the parameters of chaos. Additionally, the proposed image encryption technique has been subject to extensive study of security and performance analysis by using various statistical analyses, including: key sensitivity analysis, differential analysis, key space analysis, speed performance, and statistical test analysis. According to the results, the proposed technique is perfectly suitable for use in securing the images. The following test measurements (MSE, PSNR, and EQ, MD, SC, NC, AD, SNR, SIM, MAE, time, CC, entropy, and histograms) have shown that the proposed method delivers strong results. The proposed solution improves the security evaluation measures such as NCPR and entropy, and reduces the processing time. The proposed solution focuses on reducing the total processing time for the encryption and decryption process while improving security during the transmission process. The avalanche effect is one of the important characteristics to be considered when designing the S-Boxes, and the best value for the AC is 0.5; thereby, Table 7 proved that the proposed system achieved the AC value required because, when one bit has been changed, this leads to a change in more than half of the output. Furthermore, the proposed method for the S-Box generation process has passed all the performance analysis criteria. Finally, this solution also provides a fast security system for the surgical that helps both local and remote surgeons with secure real-time communication. The complexity of this work needs to know the chaotic method used, the values of the chaotic parameters, the S-Box creation method, and which of these methods was used in the encryption process. In future work, some other effective S-Box measurements will be checked such as nonlinearity, delta uniformity, and algebraic immunity.

## Figures and Tables

**Table 1:** The entropy values of the cipher images.

Image	Entropy
Peper1	7.998
Baboon	7.998
Ba	7.999
Tiger	7.994
Imageswb	7.990
Lena	7.991
Street	7.993
Imageswb-gif	7.991
Imageswb-png	7.996
1	7.999
2	7.997
3	7.993
4	7.998
5	7.999
6	7.994
7	7.996
8	7.998

**Table 2:** The chi-square test of images.

Image Name	Plain Image	Encrypted Image	Result
Peper	396,638.7	249.7	Pass
baboon	342,421.2	230.8	Pass
Tiger	1,923,225.8	210.1	Pass
Lena	691,867.9	200.01	Pass
Imageswb-gif	329,199.3	211.2	Pass
Street	412,865.8	219.9	Pass

**Table 3:** Time consumption for the encryption, decryption process, correlation coefficient ratio between plain images and their corresponding cipher images, and NPCR of different color components.

Image	Size	Encryption Time (s)	Correlation Coefficient	NCPR r	NCPR g	NCPR b
Peper1	225 × 225	9	−0.0002	0.992	0.997	0.997
Baboon	243 × 208	10	−0.0002	0.991	0.991	0.995
Ba	256 × 256	11	−0.0002	0.993	0.994	0.996
Tiger	225 × 225	9	−0.0001	0.997	0.996	0.997
Imageswb	225 × 225	6	−0.0001	0.993	0.987	0.996
Lena	144 × 115	1.3	−0.0001	0.992	0.991	0.997
Street	284 × 177	10.2	−0.0002	0.997	0.994	0.996
Imageswbgif	225 × 225	9	−0.0004	0.997	1	0.996
Imageswbpng	225 × 225	11	−0.0002	0.993	0.998	0.996
1	156 × 144	1.4	−0.0001	0.998	0.991	0.993
2	139 × 138	0.9	−0.0003	0.995	0.997	0.998
3	139 × 145	0.9	−0.0001	0.995	0.993	0.991
4	139 × 145	0.8	−0.0001	0.993	0.998	0.995
5	142 × 142	0.9	−0.0002	0.997	0.992	0.991
6	141 × 124	0.7	−0.0001	0.994	0.991	0.998
7	136 × 135	0.5	−0.0002	0.997	0.994	0.996
8	141 × 139	0.8	−0.0001	0.992	0.992	0.996

**Table 4:** The average differences between the created S-Boxes (1, 2, and 3).

	S-Box1	S-Box2	S-Box3
S-Box1	×	100%	99%
S-Box2	100%	×	99%
S-Box3	99%	99%	×

**Table 5:** Comparison between the proposed method and the previous works for the binary output of S-Boxes to compute balanced criteria.

Words	ABHKEF31	ZXQRASPK	01234567
**Research Paper**	**BINARRY**	**BINARRY**	**BINARRY**
[35]	01110110101111010 00101111001011010 11111111111101110 1001010001000	10010111100000001 00000101100101101 11011011111010101 010001000110	00001000100010001 10010111101001011 01011111010100011 0100001011000
[36]	01100101100000111 00101110100111000 10111000100100101 0110111011011	10101100001111110 00010111010111101 10010101110110111 1000001001110	11001011011100001 10100011010110110 11100100110011010 0011111110111
Proposed method	01000011101011001 00010111011011110 11101101100000000 0011001101110	00011011011100000 01001111001010001 00001101111011011 1001010110111	10100111011011100 11010010000011000 11111101110101001 0111001111001

**Table 6:** Comparison of the output of balance criteria using the proposed S-Box generation technique and previous works.

Words	ABHKEF31		ZXQRASPK		01234567		Average
**Research Paper**	**No. of 0s**	**No. of 1’s**	**No. of 0s**	**No. of 1’s**	**No. of 0s**	**No. of 1’s**	
[35]	24	40	33	31	36	28	93.23%
[36]	31	33	28	36	27	37	94.79%
Proposed method	32	32	31	33	28	36	97.40%

**Table 7:** Comparisons of the avalanche effect of avalanche effect of A–Z using S-BOX1 of the proposal and traditional methods.

Actual Data	65	66	67	68	69	70	71	72	73	74	75	76	77
	78	79	80	81	82	83	84	85	86	87	88	89	90
[35]	0.5	0.25	0.25	0.5	0.5	0.625	0.625	0.75	0.75	0.5	0.5	0.625	0.625
	0.375	0.375	0.375	0.375	0.375	0.375	0.25	0.25	0.625	0.625	0.375	0.375	0.625
[36]	0.25	0.625	0.625	0.625	0.625	0.5	0.5	0.375	0.375	0.375	0.375	0.5	0.5
	0.5	0.5	0.875	0.875	0.625	0.625	0.125	0.125	0.625	0.625	0.875	0.875	0.375
Proposed	0.125	0.75	0.25	0.5	0.875	0.375	0.625	0.5	0.625	0.5	0.75	0.25	0.5
	0.75	0.375	0.25	0.375	0.5	0.25	0.125	0.25	0.375	0.625	0.5	0.5	0.5

**Table 8:** Comparison between then proposed method and the previous works for the binary output of S-Boxes to compute balanced criteria.

Research Paper	Min AC	Max AC	AVG AC
[35]	0.25	0.75	0.562
[36]	0.125	0.875	0.5
Proposed	0.125	0.875	0.5

**Table 9:** Nonlinearity comparison.

S-Box Method	Max	Min	Average
Proposed	112	106	109
Ref. [46]	110	96	104
Ref. [47]	106	96	102.5
Ref. [48]	110	106	108
Ref. [49]	108	108	108

**Table 10:** Comparison of the proposed algorithm by NIST tests.

Test Name	Proposed Method	Ref. [52]	Status
Frequency	0.75	0.81	Succeed
block-frequency	1	0.65	Succeed
cumulative-sums	0.86	0.87	Succeed
Runs	0.94	0.73	Succeed
longest-run	1	0.27	Succeed
Rank	0.74	0.72	Succeed
Fft	1	0.66	Succeed
nonperiodic-templates	0.99	0.99	Succeed
overlapping-templates	1	0.60	Succeed
Universal	1	0.55	Succeed
Apen	1	0.37	Succeed
Serial	0.98	1	Succeed
lempel-ziv	0.97	0.06	Succeed
linear-complexity	1	0.79	Succeed
Random-excursions variant	0.96	0.97	Succeed
Random-excursions	0.81	0.77	Succeed

**Figure A1:**
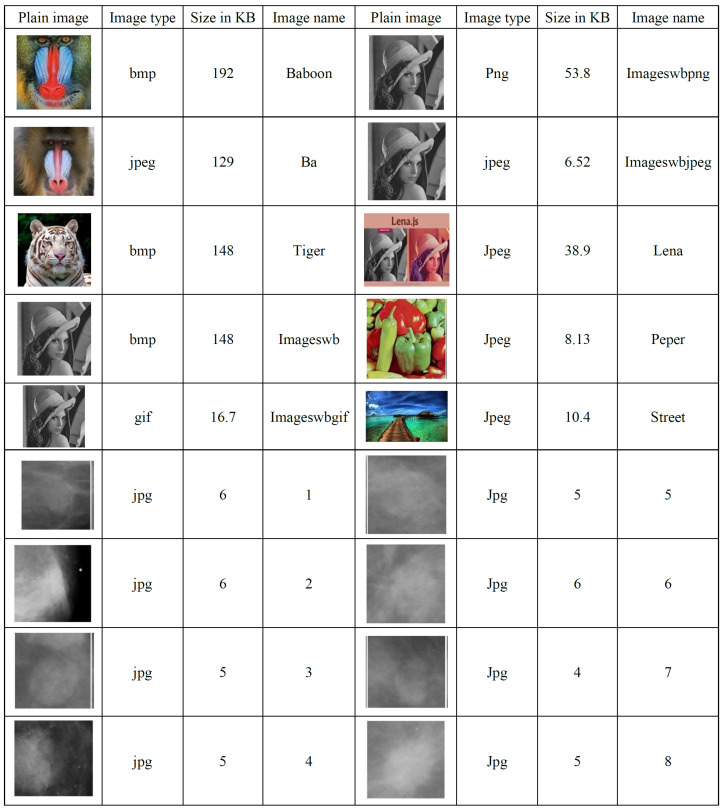
The set of tested images.

## Data Availability

The [Data type] data used to support the findings of this study are available from the corresponding author upon request. The [Data type] data used to support the findings of this study are not static and not found on any free repository. This study can be used for any [Data type] not limited to the specific resource. It can be used for any image types.

## Appendix A

The set of images included in the conducted experiments are presented in Figure A1.
